# Chromobacterium haemolyticum infection from hot springs near Yellowstone National Park: a case report

**DOI:** 10.1186/s13256-023-04005-w

**Published:** 2023-06-23

**Authors:** Kyle Patterson, Katie Suleta, Sean Shearen, Kenneth Chapman

**Affiliations:** 1 Eastern Idaho Regional Medical Center, 3100 Channing Way, Idaho Falls, ID 83404 USA; 2 HCA Healthcare, 4900 S. Monaco St., Denver, CO 80237 USA

**Keywords:** Chromobacterium, Chromobacterium haemolyticum, Case report

## Abstract

**Background:**

Chromobacterium haemolyticum is a gram-negative anaerobic sporulated rod and was only first identified in 2008. It is very rare in people with only a handful of cases having been diagnosed around the world.

**Case presentation:**

After suffering a fall near Yellowstone National Park, a white male patient in his 50 s presented to a hospital in Eastern Idaho. With many unexplained symptoms, several changes in patient stability and recovery, over a course of 18 days in the hospital, the infecting organism could not be easily identified. Labs in the hospital, state, and eventually outside of the state were consulted for pathogen identification, which was only accomplished after the patient was discharged.

**Conclusions:**

To our knowledge, this is only the seven reported human infection with Chromobacterium haemolyticum. This bacterium is difficult to identify and may be occur in rural areas without the proper testing facilities to quickly identify the pathogen, which is essential to timely treatment.

## Background

Chromobacterium haemolyticum is a gram-negative anaerobic sporulated rod. It has been found in tropical and subtropical bodies of water [1, 2]. It causes bacillary hemoglobinuria (i.e., red water disease) most often in cattle though occasionally in sheep, horses, pigs, and elk and is usually fatal [3]. C. haemolyticum produces several toxins including: phospholipase (beta toxin), tropomyosinase (eta toxin), and a lipase (theta toxin) [3].

Most human infections with chromobacterium are with violaceum [4]. It is important to differentiate between the two as C. haemolyticum displays more resistance to antimicrobial drugs than C. violaceum [5, 6]. However, infection with C. haemolyticum was first described in 2008 [5]. There have been seven reported human infections with C. haemolyticum [5–11]. These infections have occurred over a wide geographical range, including several in Japan, one in Thailand, and two in the United States (Ohio and Texas). Of these cases, multiple conditions have been reported including necrotizing fasciitis with bacteremia, pneumonia, proctocolitis, pediatric bacteremia, and meningitis [6–11]. Here, we present a case of infection with C. haemolyticum discovered in a patient in Idaho, near Yellowstone National Park.

## Case presentation

A white male patient in his fifties presented to a hospital in Eastern Idaho via helicopter after traumatic fall at Yellowstone National Park. During a hike, he slipped on wet rocks and fell six feet into a natural hot spring where he remained for eight hours while he awaited rescue. When the patient fell, he suffered a left knee laceration and left closed femur fracture, necessitating airlift. X-ray imaging in the emergency department confirmed a closed, displaced left femoral diaphyseal fracture later repaired with same-day open reduction and internal fixation (ORIF). The patient requested to be discharged home in the immediately postoperative period, however due to a drop in hemoglobin from 12.0 g/dL to 9.4 g/dL, it was recommended that the patient remain in the hospital. Two days postoperatively, the patient exhibited leukocytosis, differential diagnosis including postoperative elevation versus sepsis. There was a further decrease in his hemoglobin down to 8.0 g/dL on repeat check several hours later despite no hematoma evident on physical exam, and the patient subsequently developed a fever of 103 °F (39.4 °C) and tachycardia up to 130 beats per min. Stat orders were placed for blood cultures, complete blood count with differential, and lactate, with the left thigh noted to be firm in comparison to the surrounding tissue. Antibiotic treatment with Cefalexin was initiated at that time. By day five, the patient was noted to have polydipsia, drinking in excess of eight liters per day, and reported feeling “loopy.” Labs demonstrated hyponatremia, hypokalemia, and a hemoglobin of 7.3 g/dL, C-reactive protein of nearly 30 mg/dL, and persistence of tachycardia and fever despite a drop in his white blood cell count from 14.4 K/mm^3^ to 8.5 K/mm^3^. Blood cultures demonstrated growth of gram negative rods in four of four bottles with further identification pending, however the patient’s clinical deterioration provided sufficient evidence for the infectious disease specialty service to broaden the antibiotics from Cefalexin to Cefepime and Metronidazole. During the sixth night, repeat hemoglobin of 6.9 g/dL and suspicion of active bleeding prompted transfusion of two units of blood. Due to limitations of the in-house lab, the isolated pathogen was unable to be identified, although it was determined that the organism was a non-lactose fermenting, non-*Aeromonas* gram negative rod resistant to Cefepime. The culture was sent to a separate lab for definitive identification. The patient was transitioned to 750 mg of Levofloxacin daily for 10 days. On day 8, the patient was afebrile with improvements in heart rate, blood pressure, and hemoglobin, however white blood cell count remained elevated at 13.6 K/mm^3^ and left thigh remained firm with edematous. Pain and edema continued to increase and computed tomography scan without contrast of the lower extremity demonstrated subcutaneous edema throughout the soft tissues of the left thigh. On day 9, the patient continued to exhibit increased lower extremity edema and antibiotics were switched from Levofloxacin to Meropenem at the recommendation of the infectious disease service. Despite broadened antibiotic coverage and attempted diuresis with intravenous furosemide, the patient experienced increased pain with passive motion of the left knee and palpation to the lateral left thigh the following day, which prompted left sided lateral fasciotomy for compartment syndrome. Surveillance cultures were obtained. Leukocytosis, heart rate, and pain decreased over the remainder of the patient’s hospital stay. With negative surveillance cultures, the patient was transitioned to oral trimethoprim/sulfamethaxazole with antibiotic course completion on day 17. The patient was discharged home on day 18. Ten days following discharge, definitive culture results identified Chromobacterium haemolyticum from samples.

## Conclusions

This case demonstrates a rare infection in humans with C. haemolyticum, complicated by severe cellulitis, compartment syndrome, and sepsis. Although similarities to other case reports involving human infection with C. haemolyticum include comparable complications after exposure to this organism in colonized water sources, this case is unique given the geographic location in the Mountain West portion of the United States. Similar themes described in other case reports include contracting the organism following trauma in bodies of water [6, 8, 11], however for the first time C. haemolyticum is described as being contracted from a natural hydrothermal spring. In the Mountain West, recreation in hot springs is not only common but also represents a component of the tourism industry in the vicinity of Yellowstone. Because the hypothesized route of entry was through wound colonization following prolonged exposure in a naturally-heated water source, this raises the question as to health impact to this geographical region if infections from this bacterium become more common given the popularity of this type of recreational activity. Furthermore, these hot springs are frequently located in rural areas, near smaller hospitals that may have limited availability of both laboratory identification and treatment of naturally drug-resistant organisms. In our case report, for instance, this bacterium was unable to be identified with the capabilities of the in-house lab even at our level II trauma center, tied for highest trauma level in the Yellowstone tri-state area. This required samples to be sent out to two other labs for definitive identification. Given that this bacterium was only first described in 2008 and the mounting number of cases that are being reported, C. haemolyticum is an important pathogen to further survey. Although it would be unreasonable to suggest that the rate of infections could climb to that sufficient enough to raise a public health emergency, public health agencies and health care institutions should be made aware of these growing number of infections that have been implicated in causing high morbidity and mortality. What would be more reasonable is setting a precedent of not only prompt send-out of unidentified specimens on culture to labs capable of identification, but also ensuring proper follow up and reporting to health agencies such as the Centers for Disease Control and Prevention (CDC). Finally, a consideration of care may be that the use of steroids or other immunosuppressive drugs and presence of chronic medical illness may worsen the prognosis of this serious infection.

## Data Availability

Not applicable.
